# Linking soil-metal concentrations with children’s blood and urine biomarkers in Syracuse, NY

**DOI:** 10.1016/j.envres.2025.121816

**Published:** 2025-05-08

**Authors:** Dustin T. Hill, Michael Petroni, Vikrant Jandev, Kestutis Bendinskas, Lynn S. Brann, James A. MacKenzie, Christopher D. Palmer, Patrick J. Parsons, Mary B. Collins, Brooks B. Gump

**Affiliations:** a Falk College of Sport and Human Dynamics, Department of Public Health, Syracuse University, Syracuse, NY, 13244, USA; b Department of Environmental Science, SUNY College of Environmental Science and Forestry, Syracuse, NY, 13210, USA; c Department of Chemistry, State University of New York College at Oswego, Oswego, NY, 13126, USA; d Department of Nutrition, Syracuse University, Syracuse, NY, 13244, USA; e Department of Biological Sciences, State University of New York College at Oswego, Oswego, NY, 13126, USA; f Laboratory of Inorganic and Nuclear Chemistry, Wadsworth Center, New York State Department of Health, Albany, NY, 12237, USA; g Department of Environmental Health Sciences, College of Integrated Health Sciences, University at Albany, Rensselaer, NY, 12144, USA; h School of Marine and Atmospheric Sciences, Sustainability Studies Division, Stony Brook University, Stony Brook, NY, USA; i Institute for Advanced Computational Science, Stony Brook University, Stony Brook, NY, USA

**Keywords:** Soil-metals, Children, Environmental health, Exposome, Blood lead, Urine metal biomarker

## Abstract

This study investigates the relationship between trace element concentrations of lead (Pb), arsenic (As), cobalt (Co), manganese (Mn), molybdenum (Mo), and mercury (Hg) in soil on blood and urine biomarkers in a cohort of children from Syracuse, New York. The cohort data were collected as part of the Environmental Exposures and Child Health Outcomes project investigating the body burden of trace metals in children and where those metals come from. This study specifically investigates exposure to trace metals in soil. Cohort data were collected between 2013 and 2017 and soil samples were obtained from a separate study conducted in 2003–2004. We used linear and spatial-error models to test if daily dose exposure to soil concentrations of trace metals were associated with blood and urine levels of those same metals. We controlled for race, gender, socioeconomic status (index calculated from income and parent education level), age, body mass index, diet (index of daily dietary patterns of different food groups), smoking status of parents, distance to nearest highway, and air concentration of metal pollution. We found that a one standard deviation increase in soil-Pb may be associated with a 12 percent (95 % CI [2.5 %, 21 %]), rise in blood-Pb, and a one standard deviation increase in nearby soil-Hg levels may be associated with a 22.5 percent (90 % CI [1 %–49 %]) rise in blood-Hg. On average, participants had 2.5 μg/dL (95 % CI [1.6 μg/dL, 3.4 μg/dL]) higher blood-Pb levels for every 1000 mg/kg of soil-Pb. Participants also had 1.5 μg/g (95 % CI [0.78 μg/g, 2.2 μg/g]) creatinine higher adjusted urine-Co levels for every 1 μg/kg/day of soil-Co exposure with 10 percent variance explained by soil-Co dose (R^2^ = 0.1). These results highlight that soil-metal concentrations can pose a risk to child health even when accounting for other exposures.

## 1. Introduction

Toxic heavy metal exposures pose an urgent challenge to public health. This is especially true for children, where these exposures have caused developmental delays (Al-Saleh et al., 2020; Kao et al., 2023), neurocognitive disorders (Heng et al., 2022), behavioral disorders (Sanders et al., 2015), respiratory problems (Chowdhury et al., 2018; Liu et al., 2022; Skalny et al., 2020), cancer, and cardiovascular diseases (Hsueh et al., 2017). Due to rapid neurodevelopment, children are particularly susceptible to the detrimental health effects of environmental exposures (Heng et al., 2022). The most well documented effect of childhood heavy metal exposure is from lead (Pb) where higher levels of childhood exposure have been repeatedly tied to poor neurodevelopment, including decreased IQ, attention span, and educational attainment (Heng et al., 2022; Lidsky and Schneider, 2003).

Children can be exposed to toxic metals like Pb from a variety of pathways including dietary intake from contaminated foods (Davis et al., 2014; Gump et al., 2020), airborne metal particles from industrial releases (Wu et al., 2019), parental exposures (Kumar, 2004), and from the soil (Kormoker et al., 2021; Tchounwou et al., 2012). Elements that might be present in soil include Pb, cobalt (Co), manganese (Mn), mercury (Hg), molybdenum (Mo), and the metalloid arsenic (As). Their presence in soil might pose a risk for human health when they are bioavailable (Naidu et al., 2008) and if they are in sufficient quantity to be hazardous. Bioavailability in soil is when there is sufficient concentration of a substance to pose risk of absorption if exposed, and the substance is environmentally available, meaning that it can come into contact with a person and is not trapped deeper in the soil (Kim et al., 2015). US EPA soil-Pb standards for outdoor playable areas are recommended not to exceed 400 mg/kg as of 2023 (CDC). Metal toxicants can find their way into surface soils through deposition from air pollution, which was a pathway from leaded gasoline until its ban in 1975 that has left a legacy of Pb soil contamination for years (Mielke et al., 2010, 2017). Metals and metalloids also have found their way into the soil from historic use of insecticides containing these elements (Wagner et al., 2003). In addition, landfilling of toxicant containing compounds is another source with toxicants spreading beyond landfills from runoff (Remon et al., 2005). Some metals can remain bioavailable in the soil for many years, and while difficult to measure the length of time, some have speculated that metals could be bioavailable for decades (Kim et al., 2015). Pathways for soil-metals absorption in children include ingestion (Ljung et al., 2006) and inhalation from aerosolized soil during dry seasons when soil becomes airborne dust (He et al., 2020). Exposure to metals during childhood, even at low levels, can have negative impacts on human health including impacts on the cardiovascular system (Pan et al., 2024; Solenkova et al., 2014). Early life exposure is particularly serious for many elements that can bioaccumulate in the body causing long-term health effects (Jan et al., 2011). It is likely that geographic, cultural, and historical differences create unique regional patterns for the relative contribution of different exposure sources, suggesting that effective interventions will likely need to be region-specific.

Estimates for daily intake of soil for children is 26 mg per day (Stanek et al., 2012). Zahran and colleagues (Zahran et al., 2013) explored pathways of Pb exposure via airborne dust resuspension by first demonstrating how airborne Pb levels correspond with airborne soil levels corroborating a soil-air pathway, and second, demonstrating that air Pb is a significant correlate of child blood-Pb levels regardless of age. Their findings call into question the federal government’s focus on Pb-based paint as an effective mitigation strategy, as soil-Pb ingested via inhalation and dust contact leave parents relatively helpless to address their child’s exposure. In a cohort of 75 children residing in a Chinese industrial city, Wu and colleagues (Wu et al., 2020) identified a slight positive correlation between blood Pb levels and bioavailable Pb in soils with samples taken within 25 m at participants’ homes. Bradham and colleagues (Bradham et al., 2017) investigated the relationship between blood-Pb-levels and Pb soil contamination in a cohort of children living in Philadelphia and found that per 1000 mg/kg of Pb in soil, blood-Pb-levels rose by between 1.3 and 1.5 μg/dL. For the other metals of interest in the present study, there are more mixed results in the literature with respect to how soil-metals might impact levels within humans. Specifically, soil-As was found to be the primary source of exposure for a group of children in Australia that lived near a gold mine (Martin et al., 2013) and among a general survey of residents near previously mined areas (Hinwood et al., 2004) where a significant correlation between soil-As and urine-As concentration was observed. However, some studies have found no effect from exposure to soil-As levels suggesting that results are mixed or perhaps reflect shorter exposure periods (Tsuji et al., 2005). Co contaminated soils from smelting activities have also been linked to higher Co in children in Africa (Banza et al., 2009; Cheyns et al., 2014). Our study builds on these findings with innovation in the use of a daily dose model in addition to simple proximity measures.

We explore the association between six trace elements measured in the soil as a potential path of exposure in a cohort of children aged 9 to 11. The elements measured were Pb, As, Co, Mn, Hg, Mo. These metals were selected because they had matching biomarker data (blood or urine) with soil-metal data from a separate project, and these metals are known to cause health problems. Our research question is: do children living in areas with higher levels of metal toxicants in the soil have higher levels of those same metals in their bodies? We hypothesize that children living in areas with higher soil-metal levels will have higher levels of those metals in their bodies measured in blood and urine samples even when accounting for confounding variables and other exposure routes (e.g., air pollution). Our objectives are to link soil-metals found near the homes of our cohort to the metal levels found in the children’s blood and urine, and to use proximity and daily dose models to estimate the effect of soil metals on children’s body burden of those same metals.

## 2. Methods

### 2.1. Setting and cohort data

Historically, Syracuse, New York (NY), is known to have numerous sources of Pb, Hg, and other metals released into the air, water and in landfills (Honeywell & Onondaga Lake: A Timeline). Given this heightened exposure risk, many environmental and public health investigations have occurred since the 1970s with most of them focusing on childhood blood-Pb-levels (Griffith et al., 1998; Johnson and Bretsch, 2002). One such investigation, the “Environmental Exposures and Child Health Outcomes” (EECHO) (Gump et al., 2017) recruited children aged 9 to 11 beginning in 2013 and continuing until 2017 with roughly 60 new participants enrolled each year. This resulted in 300 total enrolled children (five years times 60 participants per year); however, after excluding participants due to missing data or dropping from the study, the final cohort size was 281. To be included, children self-identified as either White or Black (confirmed with parental report) and did not have any medical conditions that would prevent enrollment. Each participant’s home address was geocoded to match with spatial covariates. All participants and their guardians provided consent and the study was approved by the institutional review boards of Syracuse University and Upstate Medical University.

### 2.2. Biological variables – urine and blood metal concentration methods

A 5 mL venous blood sample was collected by a certified phlebotomist into a plastic lavender top (EDTA anticoagulant) tube, which was pre-certified by the analyzing laboratory for measurements of Pb and Hg. Pre-certification of blood collection tubes for trace element analysis by the analyzing laboratory is a quality assurance practice recommended by the Clinical Laboratory Standards Institute (CLSI) for blood lead (CSLI, 2024) and other trace elements (Esernio-Jenssen et al., 1999). Immediately after collection, the blood samples were inverted to ensure thorough mixing with EDTA, placed on ice and then aliquots transferred into cryovials certified for trace element analysis by the testing laboratory and frozen at −80 °C. Whole blood samples were shipped to the Laboratory of Inorganic and Nuclear Chemistry at the NY State Department of Health’s Wadsworth Center, Albany, NY for trace element analysis. The blood samples were analyzed for trace elements using a method optimized for a Thermo Xseries2 Inductively Coupled Plasma-Mass Spectrometer (ICP-MS; Thermo Fisher Scientific, MA) (Palmer et al., 2006). The ICP-MS instrument was calibrated for each element using matrix-matched calibration standards traceable to the National Institute of Standards and Technology (NIST, Gaithersburg, MD) (Palmer et al., 2006). Method accuracy was assured by analyzing NIST Standard Reference Material (SRM) 955c Toxic Elements in Caprine Blood, and method performance assessed via successful participation in four international proficiency testing programs for trace elements. Three blood-based quality control materials were analyzed in each run. Details of this well validated method has been previously described (Gump et al., 2017; Castro et al., 2019; Hill et al., 2021). There were four participants with blood Hg below the method limit of detection (LOD <0.03 μg/L); for those data points, we substituted with LOD/2. The LOD for Pb was <0.07 μg/dL.

Urine specimens were collected using supplies certified by the lab for trace element analysis and stored at −80 °C before shipping on dry ice to the same lab for analysis. Urine samples were analyzed by the same laboratory at Wadsworth using a well validated method optimized for a PerkinElmer^®^ NexION^®^ 300D ICP-MS instrument and calibrated with matrix-matched calibration standards traceable to NIST. Method accuracy was assured by analyzing NIST SRM 2668 Toxic Elements in Frozen Human Urine, and method performance assessed via successful participation in four international proficiency testing programs for trace elements. The method LOD for each element was 0.31 μg/L for Pb, 0.059 μg/L for As, 0.031 μg/L for Co, 0.082 μg/L for Mn, and 0.45 μg/L for Mo. Four levels of internal QC were analyzed with acceptable CVs (<15 %). Additionally, two levels of NIH CHEAR RMs were analyzed with acceptable performance (±15 %) and precision (CV < 15 %). Due to high inter-association between urinary metal concentrations, results were adjusted for creatinine, i.e., expressed in μg/g creatinine. Samples were diluted 20-fold in distilled deionized water and a duplicate measurement was performed using an ENZO^®^ Creatinine Colorimetric Detection Kit in a modified Jaffe reaction with standards and blank on each 96-well plate. The linear fit was used and the average value (after adjustment for dilution) was 131 μg/dL. The intra-assay precision was 1.0 %, inter-assay precision was 3.4 %. All readings were above the manufacturer’s LOD of 0.042 mg/dL. Further details on the analysis of urine specimens are detailed in Hill et al. (2023).

### 2.3. Soil data

As part of a prior and separate research project (Griffith et al., 2009), parcel soil and house dust sample data collection fieldwork were conducted across the City of Syracuse over three years. A total of 3627 useful soil samples were collected, mostly during the summers of 2003 and 2004. A NITON XL-700-series X-ray fluorescence (XRF) instrument was used in a chemistry laboratory to measure a suite of trace elements including—Pb, As, Co, Mn, Hg, and Mo—in these soil samples, based on 120-s testing time and NIST SRM 2711 of Montana Soil to monitor accuracy. Published detection limits relevant to this protocol were used to screen these data. The outcome was 3297 useful soil samples. Each soil sample location was geocoded with a GPS unit. Further description of the data and methods has been published by Griffith et al. (2009). While these data were not collected during the EECHO cohort study years, they were the best available soil-metal data, and metals can remain available in soil for decades (Kim et al., 2015) meaning that the cohort was likely exposed to similar levels. Linking the soil data to participants was done using several methods including three for proximity (closest soil sample to the participant home) and three for interpolation, described below.

### 2.4. Proximity variables

Three methods were tested for assigning soil testing data to the EECHO Syracuse cohort based on proximity to soil sample collection. For the first method, participants were assigned concentration estimates based on the closest sample location to their home residence out of all samples within 90 m of their residence. For the second measure, buffer estimates were generated by averaging the samples within 90 m and also 275 m of the home. The advantage of using a buffer was that it reduced the variability and potential error related to assigning a child to a soil estimate that was substantially higher or lower than the area average and helps reduce error from child movement patterns. The third proximity measure was the maximum detected level of a sample within 90 m and 275 m of the home. This method had the advantage of being the most liberal with regard to exposure, avoiding any low sample results biasing exposure away from potential nearby high soil-metal concentrations. This method was also limited by the assumption that that maximum measured value was representative of broader exposure. Each participant had one value assigned to them for each proximity measure (e.g., one maximum value for 90 m).

### 2.5. Interpolation

In addition to proximity exposure variables, we also used spatial interpolation to assign soil values across the larger spaces between participant homes that did not have measured soil concentration data. We used the methods described by Wu et al. (2011) for spatially interpolating soil data including methods for skewness. We estimated three interpolated soil values for each method to include in our comparisons for soil exposure. First, we created a grid across the study area of 50 m^2^ units providing 43,818 data points to spatially interpolate over. The first interpolation method we used was Ordinary Kriging (OK), which is a linear approximation method for spatial data that assumes a constant mean value across a region (Wackernagel and Wackernagel, 2003). The second method we used was triangular irregular surface (TIN) which takes a nonlinear approach to interpolation and assumes irregularity of the surface (LEE, 1991).The third method we used was proposed by Wu et al. (2011) for skewed data. This method estimates a combined value that sums a TIN estimate calculated from the outlier observations only with an OK estimate made from the observed data without the outliers. The method (hereafter noted as TIN + OK split dataset) assumes that the outlier data comes from a separate distribution and therefore two methods for interpolation are needed to best capture the expected values (Wu et al., 2011). We also conducted two other variations of the combined TIN and OK method. In addition to combining the values from the split datasets calculating the TIN and OK separately for the outlier dataset and the normally distributed dataset, we also calculated the values for the combined dataset then summed them (hereafter known as TIN + OK full dataset). Second, we added a step where prior to Kriging, we log-transformed the dataset without the outliers and used that in the calculation (hereafter known as TIN + LG OK, split dataset). Each participant had one value assigned to them for each interpolated value (e.g., OK, TIN and TIN + OK); these were the values that aligned with their home after the interpolation step.

### 2.6. Dose calculations

Since contaminants in soil can be exposed to individuals in different ways including ingestion and inhalation of soil dust (Stanek et al., 2012), we included an exposure dose estimate for each participant based on their potential intake per day. We used the exposure dose equation (Equation (1)) published by The Agency for Toxic Substances and Disease Registry (ATSDR) (ATSDR):

Equation 1 $$\text{Exposure}\;\text{Dose}(D)=\frac{C*IR*EF*CF}{BW}$$

*C* = assigned soil concentration value (mg/kg)

*IR* = Media-specific intake rate (60 mg/day)

*EF* = Exposure factor

*CF* = conversion factor (10^−6^ kg/mg)

*BW* = body weight (kg)

In Equation (1), we assigned the soil concentration value to each participant from our interpolation steps that had the lowest root mean squared error when compared to the observed soil sample data (see supplemental material for full table). We assume that this value represents the most likely average concentration that the child was exposed to. We include the media-specific intake rate published by the ATSDR (ATSDR), an exposure factor (EF), and the body weight of the child. The EF (Equation (2)) is the ratio of the product of all years of potential exposure multiplied by the soil concentration assigned to the participant as compared to the nonresidence years of exposure multiplied by the average soil concentration for the City of Syracuse (US EPA NATIONAL CENTER FOR ENVIRONMENTAL ASSESSMENT and W. D).

Equation 2 $$\text{Exposure}\;\text{factor}(EF)=\frac{EY}{AY}$$

*EY* = residence years*EC + nonresidence years*EC

*EC* = exposure concentration

*AY* = child age*average concentration

The EF makes a few assumptions about each participant. First, we assumed that the child’s primary exposure came from the soil concentration values nearest their home. This of course leaves out potential exposure from schools, parks, or previous residences. Second, we modified the EF using the average concentration for the metal for the entire city assuming that the mean concentration across the larger geography can approximate the average exposure the child might encounter in other locations than their home. Third, we did not have previous residences for the children, so for years with residence elsewhere, we also substituted the city soil-metal average as the exposure. We conducted a sensitivity analysis for years of residence finding that the longer time a child spent at the same residence, the stronger the statistical associations with the mean imputation still having a significant effect. See the supplemental material for more details on these results.

### 2.7. Covariates

We included several covariates to control for confounding explanations of differing levels of blood and urine metals in participants. Participant characteristics that were added to our models included socioeconomic status (SES), body mass index percentile, and race. We also included the age of the participant as others have found significant variation in the biomarkers we examined due to age (Bradham et al., 2017). We also included a variable for the maximum reported temperature on the day of the blood draw. Previous research has shown that blood-metal levels (specifically Pb) can vary by climate and season (Johnson et al., 1996; Laidlaw et al., 2005). Additionally, sources of metals in the body can come from exposure to Pb dust in vacant homes (Castro et al., 2019), diet (Davis et al., 2014; Gump et al., 2020), and air pollution (Hill et al., 2021). For home vacancy, we estimated the percentage of vacant homes in each block group using 2010 decennial census data downloaded using the tidycensus R package (Walker and Herman, 2022). We included known dietary predictors (Gump et al., 2020) and known air pollution covariates from previous analyses to control for other exposures (Hill et al., 2021). Distance to highways was also included and controls for potential soil-to-dust formulation from highway traffic and is a proxy for localized vehicle emissions.

### 2.8. Statistical analyses

We fit linear regression models for each participant’s outcome data (blood and urine-metal levels) plus covariates and soil exposure data. Each soil exposure was included in separate models to compare each method. We also tested each outcome for spatial autocorrelation using a Moran’s I. Variables that had significant autocorrelation (p value < 0.2) were refit using a spatial error model (Bivand and Piras, 2015) that includes a spatially weighted error vector (U) comprised of the spatial autoregression coefficient (lambda) and a spatial weights matrix (W) based on the point’s nearest neighbor, and a normally distributed error vector (e) detailed in equation (3).

Equation 3 Y=βX+u,u=λW+e

Covariates did not display issues of multicollinearity (no variance inflation factor >2), but there was some outlier data observed in the outcome (values outside the interquartile range times 1.5). We fit each model without outliers to ensure that the outliers were not skewing the models in any specific direction and present those results in our supplementary material. Last, to accommodate for children that did not live in the same residence their entire life, we did a sensitivity analysis for blood-Pb and urine-Co based on the years the child reported living in the same residence to explore any potential differences based on the duration of the assigned exposure values. All analyses were conducted in R version 4.1.1 (R Core Team, 2021).

## 3. Results

### 3.1. Participant and soil data

Of the originally enrolled 298 participants, 281 had home locations within 275 m of a collected soil sample and were included in the study. Further, we collected 234 urine samples and 279 blood samples from the cohort (Table 1). Of the originally collected soil samples, 3297 had measurable levels of Pb and Mn, 2922 had measurable levels of As, 2820 had measurable levels of Hg, 2143 had measurable levels of Mo, and 1883 had measurable levels of Co (Table 1).

**Fig. 1.**
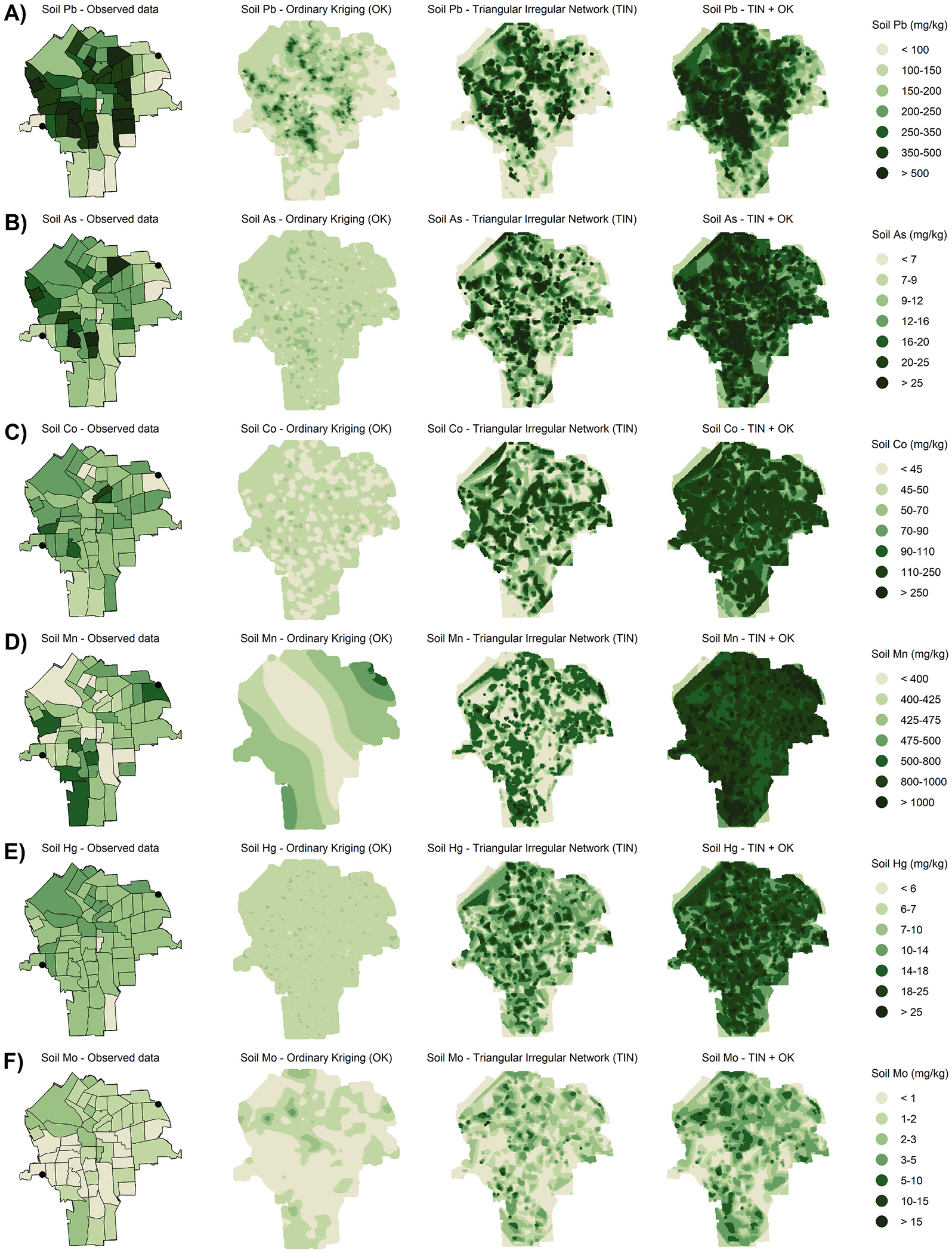
Observed and interpolated soil concentration estimates for A) Pb, B) As, C) Co, D) Mn, E) Hg, and F) Mo.

Interpolation yielded coverage of the entire City of Syracuse for an estimate of soil concentration every 50 m. Each interpolation method varied in its ability to estimate soil coverage across the city (Fig. 1). Soil-Pb was concentrated most heavily in the near West Side of Syracuse as well as the North and East parts of the city (Fig. 1). This pattern was mirrored by soil-As to a lesser extent, and soil-Mn, while soil-Co and soil-Hg were more spread out around the city (Fig. 1). Soil-Mo had some of the lowest concentrations and these were highest in the northern part of the city and the southwestern portion (Fig. 1).

### 3.2. Soil-metal and biomarker associations

When testing for associations between the proximity of metals in soil and the child’s body burden of those metals, we found significant correlation between several soil concentrations and biomarkers in the cohort. For blood-Pb and soil-Pb, there was a significant, positive correlation for the soil exposure dose metric (R = 0.344, p value < 0.01, Table 2). For urine-Pb, similar associations were found with the strongest being for the soil dose metric (R = 0.244, p value < 0.01, Table 2). For urine-Co, there was a statistically significant correlation with soil dose level (R = 0.189, p value < 0.05, Table 2). For blood-Hg, there was a statistically significant correlation with the maximum soil concentration within 275 m (R = 0.161, p value < 0.05).

Soil-metal levels remained significant predictors of blood and urine metal levels for some of the outcome variables in linear and spatial models with covariates. For blood-Pb, the dose metric and the OK soil concentrations were the most significant (p value < 0.05, Table 3). For the soil-Pb dose exposure model, a one standard deviation rise in the exposure dose was associated with a 12 percent rise in blood-Pb level (β = 0.109, p value < 0.05, 95 % CI [2.5 %, 21 %], Table 3). The average potential daily dose of Pb from soil exposure for our participants was estimated at 0.617 μg/kg of body weight/day. For the model results using proximity and interpolated variables, a one standard deviation increase in the soil-Pb levels from the OK interpolation method was associated with a rise in blood-Pb levels of 15 percent (β = 0.138, p value < 0.01, 95 % CI [5 %, 25 %], Table 3).

For Co, a one standard deviation rise in the exposure dose was associated with a 3.98 percent rise in creatinine adjusted urine Co level (β = 0.039, p value < 0.05, 95 % CI [1 %, 7.9 %], Table 3). The average potential daily dose of Co intake from soil exposure in Syracuse was estimated at 0.103 μg/kg body weight/day. Blood-Hg was significantly associated with the maximum soil-Hg concentration within 275 m (p value < 0.05). A one standard deviation increase in the soil-Hg dose was moderately associated with a 22.5 percent rise in blood-Hg levels (β = 0.203, p value < 0.1, 90 % CI [1 %, 49 %], Table 3). The average potential daily dose of Hg intake from soil exposure in Syracuse was estimated at 0.015 μg/kg body weight/day. For urine-Pb, the OK method yielded only moderately significant results (β = 0.047, p value < 0.1). No associations were found in the linear or spatial models for urine-Mo and soil-Mo levels or urine-Mn and soil-Mn levels. See supplementary materials for full regression results for all metals.

**Fig. 2.**
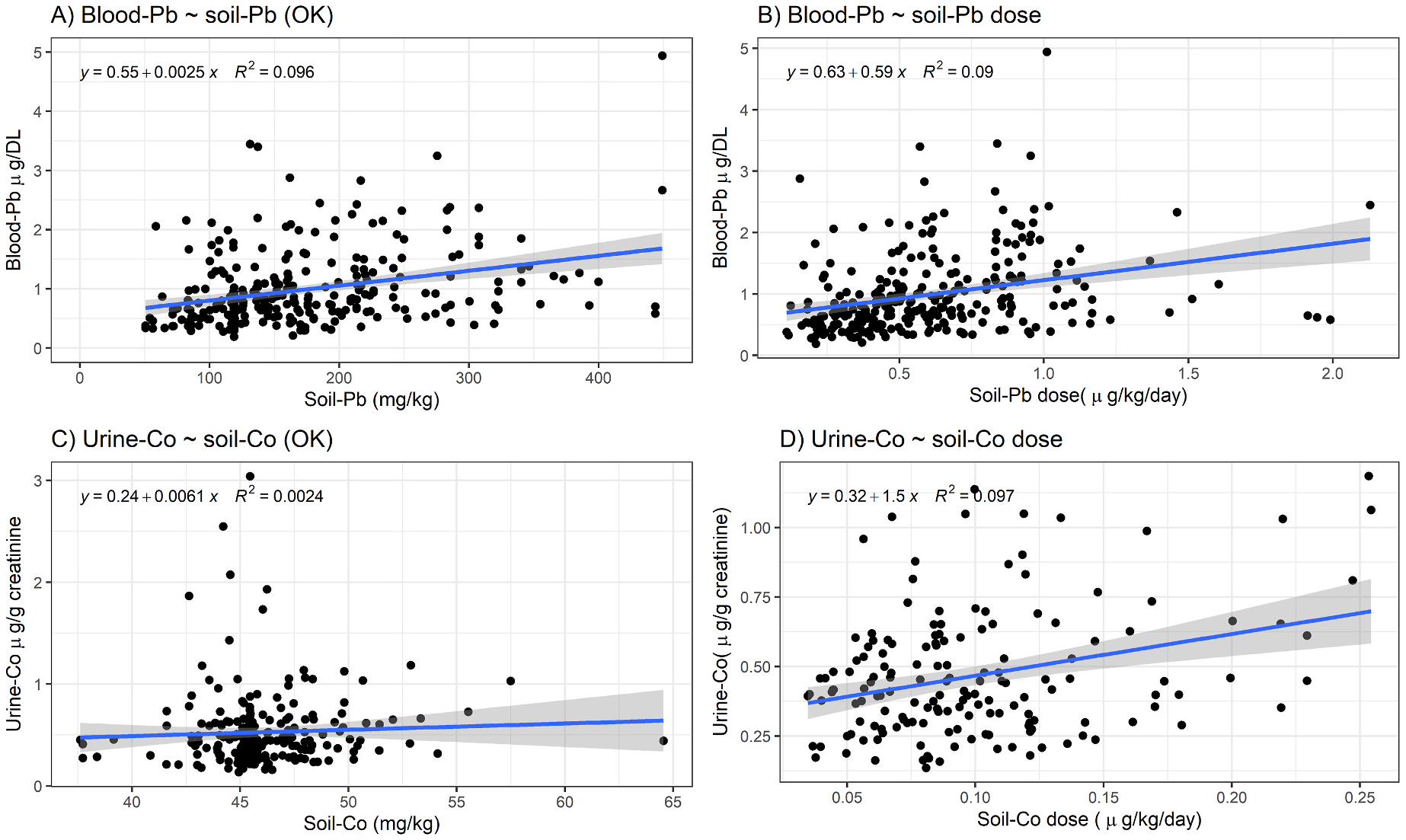
A) Linear regression for blood-Pb and OK soil-Pb (4 outliers removed). B) Linear regression results for blood-Pb and soil-Pb exposure dose (6 outliers removed). C) Linear regression results for urine-Co and soil OK soil-Co (no outliers removed). D) Linear regression results for urine-Co and soil-Co exposure dose (5 outliers removed).

The log-transformed models had the best linear fit between the soil exposure variables and the blood/urine metal outcome variables, however, we also fit models with the data on their original scales, unstandardized. We did this to contribute estimates for the change in soil concentration and its association for the change in blood/urine metal levels for the soil metrics that were statistically significant for blood-Pb, and urine-Co. Participants on average had 2.5 μg/dL (95 % CI [1.6 μg/dL, 3.4 μg/dL]) higher blood-Pb levels for every 1000 mg/kg of soil-Pb with 9.6 percent variance explained (Fig. 2a). Participants had 0.59 μg/dL (95 % CI [0.37 μg/dL, 0.82 μg/dL]) higher blood-Pb for every 1 additional μg/kg/day of soil-Pb exposure with 9 percent variance explained by soil-Pb dose (Fig. 2b). For soil-Co dose, participants had 1.5 μg/g (95 % CI [0.78 1.5 μg/g, 2.2 1.5 μg/g]) creatinine higher creatinine adjusted urine-Co levels for every 1 μg/kg/day of soil-Co exposure with 9.7 percent of the variance explained by soil-Co dose (Fig. 2d).

## 4. Discussion

### 4.1. Connections to previous studies

For soil-Pb and blood-Pb, our results expand on earlier findings by Johnson and Bretsch (2002) who reported a positive and significant association for children living in areas of higher soil-Pb levels having higher blood-Pb levels in Syracuse using data collected between 1992 and 1996. Our refined models incorporated covariates previously thought to confound soil-Pb as an exposure and includes other potential sources like diet. This suggests that soil contamination remains a source of Pb exposure in the City of Syracuse (Laidlaw et al., 2005). Comparing our results to a study by Bradham et al. (2017) who found that per 1000 μg/g of Pb in soil, blood-Pb levels rose between 1.3 and 1.5 μg/dL, our findings suggest a slightly larger effect size where per 1000 mg/kg, blood-Pb levels rose between 1.7 and 2.7 μg/dL (Fig. 2a) and our model included other exposure covariates. Further, our findings corroborate other studies that postulated that rising temperature leads to greater evapotranspiration and decreased soil moisture leading to soil dust that elevates Pb dust loading in the air and could lead to exposure through inhalation. Our findings also find inhalation of soil dust to be a pathway of exposure for other metals like Hg and Co as well as a possible route for metalloid As. In addition, the findings are particularly unique for Pb and Hg since we looked at soil exposure with other exposures in those models. For blood-Pb, the leading exposure source by effect size in our standardized model was parental smoking (β = 0.131, p value < 0.1, Table 3), followed by soil-Pb dose (β = 0.109, p value < 0.05, Table 3), vacant home density (Pb paint indicator, β = 0.108, p value < 0.05, Table 3), and total fruit in diet (β = 0.072, p value < 0.05). For blood-Hg, the leading exposure by effect size in our standardize model was Hg air pollution from industrial sources (β = 0.580, p value < 0.05, Table 3), followed by soil-Hg dose (β = 0.203, p value < 0.1, Table 3), and total protein in diet (β = 0.160, p value < 0.01, Table 3).

In addition, three of our best statistical models were based on the relationship between soil-metal dose suggesting that the does-exposure modeling approach might be among the better approaches for estimating exposure and outcome. These models have the added robustness of incorporating time spent at the home and age in ways that make the exposure estimate more realistic than just proximity.

### 4.2. Limitations

Our findings must be considered in the context of the study limitations. First, we tried to capture the entire exposome that our cohort faced but we were missing some pathways of exposure including maternal exposure, drinking water, and exposure prior to living in their residence at the time of study. The lack of previous home locations is of greatest impact on our proximity and interpolated models because they assume that exposure was constant, but it is very possible that cohort participants that moved (n = 205) had different exposures. Sensitivity tests for participants that did not live their entire life at the home found stronger associations than those living there less suggesting that more specific exposure is best when matched up with participants living in the same place the longest. The dose models did incorporate years of exposure in the equation and were likely less impacted by the residency changes. Still, having a complete residential history would likely have yielded stronger model results given the associations improved for children who did not change residence (see Supplementary Material). The use of the location nearest the home is also a limitation since any exposure occurs at all locations a person visits, which would include home and school for the cohort. Nearby soil-metal levels may not be the best proxy for the average exposure a child might face, but they were the best available measures that we had and our findings agree with previous findings that take a similar approach (Mielke et al., 2017; Bradham et al., 2017; Johnson and Bretsch, 2002; Laidlaw et al., 2005). We also did not consider the changing bioavailability of metals that could change exposure risk (Wu et al., 2020) nor did we do any modeling of additional pathways from soil to air for children that may explain some of the variation (Zahran et al., 2013). High Pb dust in summer is also of concern, and our control for seasonality was temperature. This is a crude proxy and while better measures exist, it is unlikely that there were systematic differences in the number of summers experienced by participants, aside from age, which was included in all models. Last, there is some temporal mismatch between the soil data collected between 2003 and 2004 whereas participant data collection occurred between 2013 and 2017. This means that the soil data is most relevant to the cohort for their early years (children in the study were born between 2002 and 2007), however, given that metals in soil can remain available for decades, if not longer, we foresee this as only a minor problem and the exposure matchup for early life could benefit the study given that the exposure during early life is most acute.

### 4.3. Recommendations for action

Children play in backyards, on playgrounds, and may ingest soil or inhale soil dust in the summer. There is a high degree of complexity with regards to soil-metals becoming an exposure risk including the average dose per day, requiring the right conditions to expose a person, and toxicants being near the surface to pose a risk. Our study shows that even when taking these complexities into account and accounting for other exposure points, soil-metal concentrations pose a risk for children’s health. Solutions to the problem are not easy and most of the soil-metal levels are within what is considered safe for play areas by the EPA (i.e., 400 mg/kg for soil-Pb at a child occupied facility) (CDC), which suggests that these levels might not be low enough to protect children. The EPA updated the soil-Pb level recommendation for residential properties in January 2024 to 200 mg/kg (US EPA, O.), and does not recommend gardens in soil with levels above 100 mg/kg. Our findings support efforts to reduce anthropogenic contributions of contaminants to the soil to reduce the risk of exposure even in areas where clear sources of the toxicant are not present, like a smelter or landfill. Further, cities and community organizations can help reduce exposure by recommending the use of raised beds for residential gardening, which might be essential for cities with large refugee and immigrant populations that are more likely to live in urban areas and create home gardens. Also, encouraging good hygiene practices with young children like washing hands after playing outdoors, washing footwear that is muddy or dirty, and removing footwear before entering the home may be advisable. Last, long term, low-level exposure to dust and soil-Pb might not be as aggressive at leading to Pb poisoning in children as Pb in paint, but it is still hazardous and deserves attention. Reducing Pb poisoning will take a combined approach that includes addressing Pb contamination from all potential sources, backyards included.

## Supplementary Material

1

## Figures and Tables

**Table 1:** Descriptive statistics for all variables.

Variable	n	Mean^a^	sd	Min	Median	Max
Outcome variables (urine metal levels adjusted for creatinine)						
Urine-As (μg/g creatinine	234	22.6	86.8	0.358	5.79	897.6
Urine-Co (μg/g creatinine)	234	0.528	0.361	0.135	0.447	3.04
Urine-Mn (μg/g creatinine)	234	0.263	0.718	0.009	0.159	10.3
Urine-Mo (μg/g creatinine)	234	69.3	35.0	16.8	64.2	384.1
Urine-Pb (μg/g creatinine)	234	0.597	0.938	0.022	0.38	9.52
Outcome variables (blood metal levels)						
Pb (μg/dL)	279	1.039	1.048	0.190	0.800	14.7
Hg (ng/mL)	279	0.381	0.803	0.010	0.210	11.7
Soil-metal concentrations						
As (mg/kg)	2922	13.4	34.6	0.00	9.00	1241
Co (mg/kg)	1883	67.5	52.1	0.00	56.3	504
Hg (mg/kg)	2820	8.4	5.51	0.00	8.00	40.0
Mn (mg/kg)	3297	448.7	162.341	1.00	431	1668
Mo (mg/kg)	2143	1.025	1.777	0.00	0.00	29.0
Pb (mg/kg)	3297	343.6	1443.216	1.00	127.3	56819
Other predictors and covariates						
HEI^b^ - sodium	252	3.68	2.86	0.00	3.87	10.0
HEI^b^ - total fruit	252	2.84	1.83	0.00	2.98	5.00
HEI^b^ total protein	252	4.01	1.22	0.083	4.54	5.00
BMI percentile^c^	281	68.3	30.488	0.002	78.6	99.9
Age at blood draw	281	10.5	0.907	8.992	10.4	12.1
Distance to nearest highway (m)	281	1121	667	50.5	1050	3345.8
Hg air concentration (μg/m^3^)	275	1.237E-05	0.241	3.217E-06	1.131E-05	1.811E-05
Percent of vacant homes in participant’s block group	281	0.121	0.070	0.015	0.114	0.281
SES score^d^	281	0.073	0.829	−1.56	−0.129	2.071
Max temperature on day of blood draw (°C)	281	14.3	10.87	−7.70	13.9	31.7
Gender - female	132					
Gender - male	149					
Parent does not smoke	168					
Parent smokes	112					
Race – non-Hispanic, white	117					
Race – African American	164					

Notes.

a arithmetic mean.

b HEI values are from the Healthy Eating Index and represent a score for assessing adherence to the 2015–2020 dietary guidelines.

c Body mass index (BMI) was converted to CDC growth chart percentiles.

d Socioeconomic status or SES is a unitless index and was calculated using a composite variable based on occupation, income, and education previously described in Castro et al. (2019).

**Table 2:** Pearson correlation values for each soil exposure metric and blood/urine biomarkers. Outcomes and predictors are log transformed.

	Blood-Pb	Urine-Pb	Urine-As	Urine-Co	Urine-Mn	Blood-Hg	Urine-Mo
Dose	0.344 ***	0.244 ***	0.105	0.189 **	−0.011	−0.013	0.157
TIN and OK combined	0.272 ***	0.165 **	0.076	−0.03	−0.02	0.065	0.05
Ordinary Kriging (OK)	0.326 ***	0.232 ***	0.043	0.078	−0.071	0.046	0.018
Triangular irregular network (TIN)	0.18 ***	0.03	0.052	−0.073	−0.013	0.07	−0.05
Max 275 m	0.186 ***	0.099	0.083	0.069	−0.09	0.161 **	0.031
Mean 275 m	0.231 ***	0.151 **	0.096	0.012	−0.082	0.031	0.042
Max 90 m	0.128	0.146	−0.017	0.08	0.072	−0.006	0
Mean 90 m	0.201 **	0.199 **	0.022	0.113	0.019	−0.055	0.016
Closest value	0.247 ***	0.195 **	0.085	0.145	0.105	0.133	0.029

*** p value < 0.01,

** p value < 0.05,

* p value < 0.1.

**Table 3:** Select biomarker-soil-metal model results. All models are spatial error adjusted. Coefficients are standardized. Outcome is natural log-transformed, and the multiplicative effect size on the outcome can be obtained by exponentiating the coefficient. Interpretation of coefficients using the dose model as the example. Estimate = 0.109, exp(0.109) = 1.12. A 1 SD increase in soil-Pb dose equates to an increase in blood-Pb by a factor or 1.12 or a 12 % increase. For the spatial predictors (second row) different transformations were used based on the lowest RMSE for that metal’s interpolated data when compared to the observed data.

Predictor name	Blood-Pb		Urine-As		Urine-Co		Blood-Hg	
	Dose: OK (natural log)	Ordinary Kriging (OK) (natural log)	Dose: TIN and OK combined (natural log)	TIN and OK combined (natural log)	Dose: TIN (natural log)	Triangular irregular network (TIN) (natural log + 1)	Dose: OK (not log transformed)	Ordinary Kriging (OK) (not log transformed)
	Est. (SE)	Est. (SE)	Est. (SE)	Est. (SE)	Est. (SE)	Est. (SE)	Est. (SE)	Est. (SE)
Intercept	−0.099 (0.067)	−0.089 (0.067)	2.304*** (0.114)	2.301*** (0.114)	0.366*** (0.025)	0.370*** (0.022)	4.421 (3.26)	4.359 (3.279)
Soil concentration	0.109** (0.043)	0.138*** (0.045)	0.057 (0.083)	0.063 (0.066)	0.039** (0.019)	−0.012 (0.013)	0.203* (0.123)	0.063 (0.072)
Race	−0.175** (0.082)	−0.194** (0.081)	−0.023 (0.136)	−0.003 (0.138)	0.051 (0.032)	0.049* (0.027)	−0.14 (0.161)	−0.126 (0.162)
Gender	−0.116* (0.066)	−0.131** (0.066)	−0.037 (0.125)	−0.050 (0.125)	0.064** (0.027)	0.063*** (0.024)	0.148 (0.141)	0.097 (0.139)
SES	−0.021 (0.04)	−0.011 (0.04)	−0.130* (0.071)	−0.129* (0.071)	−0.015 (0.016)	−0.016 (0.014)	−0.019 (0.08)	−0.024 (0.081)
Distance to highway	0.059 (0.038)	0.064* (0.038)	0.062 (0.065)	0.062 (0.065)	0.011 (0.014)	0.016 (0.013)	−0.022 (0.075)	−0.024 (0.076)
BMI	−0.059 (0.039)	−0.092*** (0.034)	−0.032 (0.075)	−0.057 (0.063)	−0.02 (0.017)	−0.022* (0.012)	0.15. (0.108)	0.019 (0.072)
Age	−0.061* (0.035)	−0.083** (0.034)	−0.044 (0.066)	−0.064 (0.063)	0.038*** (0.014)	0.022* (0.012)	0.068 (0.083)	−0.003 (0.075)
Parent smoking status	0.131* (0.078)	0.139* (0.077)	−0.148 (0.14)	−0.145 (0.139)	−0.063** (0.03)	−0.047* (0.027)	−0.038 (0.156)	−0.02 (0.156)
HEI total fruit	0.072** (0.034)	0.070** (0.034)	–	–	–	–	–	–
Percent vacant homes	0.108** (0.043)	0.069 (0.047)	–	–	–	–	–	–
Maximum temperature on day of sample	−0.004 (0.035)	−0.006 (0.035)	0.096. (0.064)	0.096. (0.064)	0.007 (0.014)	0.003 (0.012)	0.003 (0.073)	0.003 (0.073)
Hg air pollution (Hg models only)	–	–	–	–	–	–	0.580** (0.286)	0.574** (0.288)
HEI sodium	–	–	–	–	–	–	−0.03 (0.025)	−0.027 (0.025)
HEI total protein	–	–	–	–	–	–	0.160*** (0.06)	0.158*** (0.06)
R2	0.300	0.310	0.040	0.050	0.180	0.100	0.090	0.080
n	249	249	233	233	165	233	244	244
Moran’s I	2.807***	2.857***	0.919	0.920	1.720**	1.420*	1.436*	1.446*

*** p value < 0.01,

** p value < 0.05,

* p value < 0.1.

## Data Availability

Code used for the interpolation methods and linking to the blood and urine biomarkers is available on GitHub. https://github.com/dthill196/syracuse-soil-metal-biomarker-study. Soil data must be requested from the authors of that paper (Griffith et all) (Griffith et al., 2009). Other data are available upon reasonable request to the manuscript authors.

## Appendix A. Supplementary data

Supplementary data to this article can be found online at https://doi.org/10.1016/j.envres.2025.121816.

## Abbreviations

Pb: lead
As: arsenic
Co: Cobalt
Hg: mercury
Mn: manganese
Mo: Molybdenum
EECHO: Environmental Exposures and Child Health Outcomes
OK: ordinary Kriging
TIN: triangular irregular network
