# Supplementary material for: Linking soil-metal concentrations with children’s blood and urine biomarkers in Syracuse, NY
Source: Environ Res. Author manuscript; Available in PMC 2026 May 21. (PMC13193073; doi:10.1016/j.envres.2025.121816)
Supplement: 1 [file NIHMS2175657-supplement-1.docx]

Supplementary material

# GitHub repository and data availability

Code used for the interpolation methods and linking to the blood and urine biomarkers is available on GitHub. <https://github.com/dthill196/syracuse-soil-metal-biomarker-study>. Soil data must be requested from the authors of that paper (Griffith et all).^1^ Other data are available upon reasonable request to the manuscript authors.

# Spatial distribution of soil samples and participants

Participant data was distributed around the city with most participants enrolled from the South and near West Side of Syracuse due to focused enrolment in those zip codes. Soil samples were also distributed fairly evenly across the city with a mean of 53 samples collected in each US Census Tract with the heaviest sampling occurring in the east side of Syracuse near the university and the second highest number collected from the South Side (Supplemental Figure 1).

| 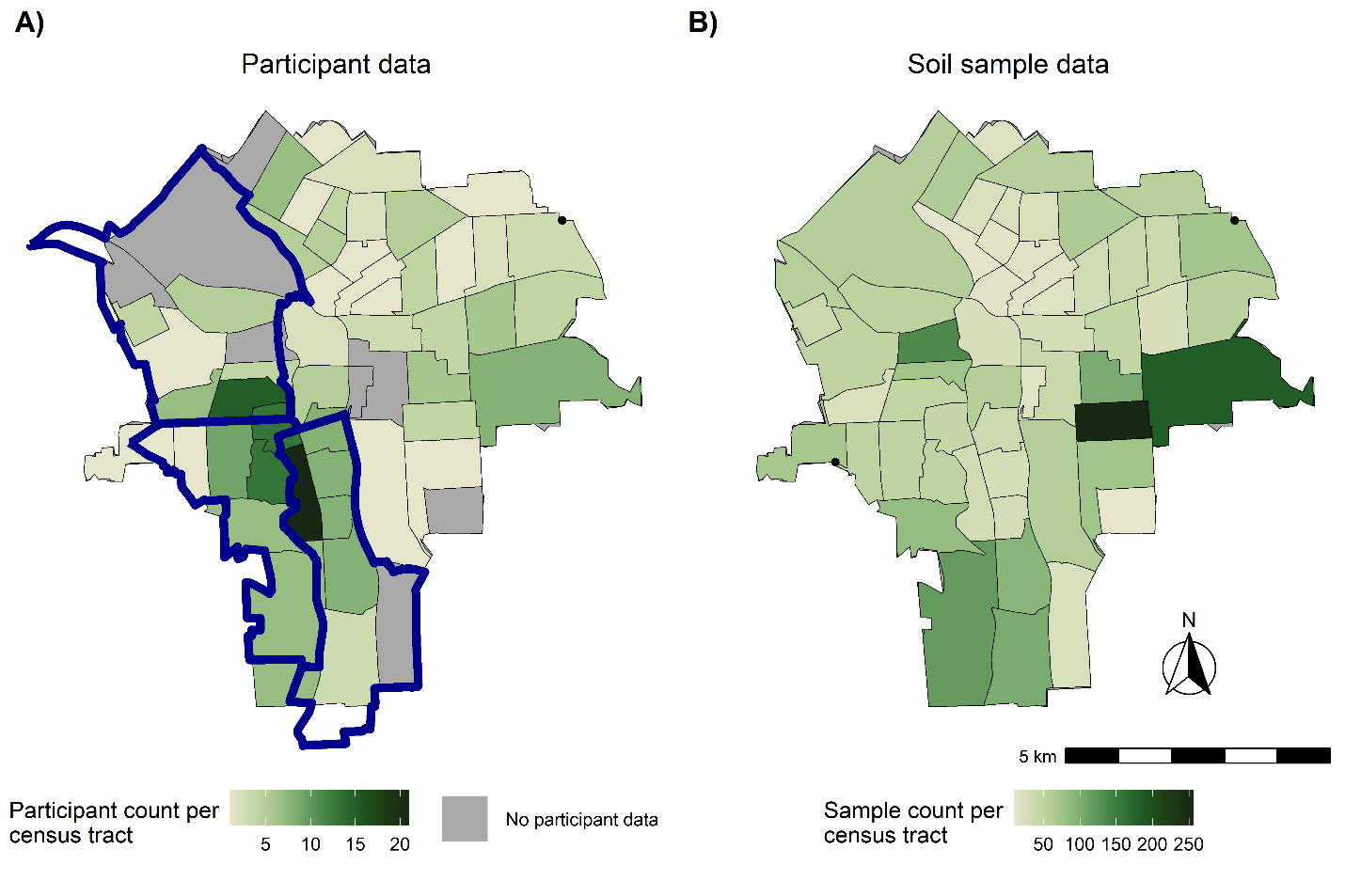 |
| --- |
| **Supplemental Figure 1**: A) Participant data were collected across Syracuse, NY. Initial enrollment was focused on the zip codes 13204, 13205, and 13207. B) Soil samples were collected throughout the city with a mean of 53 samples per census tract. |

# Soil interpolation RMSE values

When comparing interpolated values to the measured soil data, some interpolation method performed better than others. Based on the residual mean square error (RMSE), the TIN and LG OK (full dataset) method was the most closely linked to the observed soil Pb values and soil As values (lowest RMSE, Supplemental Table 1). For soil Mn, the ordinary Kriging on the log-transformed data (LG OK, full dataset) was the most accurate (Supplemental Table 1). Foil soil Co and soil Mo, the TIN method was the most accurate (Supplemental Table 1). Last, for soil Hg, the OK method was the most accurate (Supplemental Table 1).

| Supplemental Table 1: RMSE values for each interpolation method compared to the sample data. The lowest RMSE was used to select the method used in the dose-model calculation. | | | | | | |
| --- | --- | --- | --- | --- | --- | --- |
| *Interpolation method* | *Pb* | *As* | *Mn* | *Co* | *Hg* | *Mo* |
| LG OK (full dataset) | 1457.92 | 34.60 | 162.60♦ | 54.44 | 5.61 | 1.50 |
| TIN (full dataset) | 1364.18 | 32.82 | 247.94 | 49.81♦ | 5.81 | 1.25♦ |
| OK (full dataset) | 1479.25 | 34.62 | 163.78 | 51.58 | 4.99♦ | 1.49 |
| LG OK (split dataset) | 1484.20 | 34.84 | 163.20 | 54.67 | 5.54 | 1.45 |
| TIN (split dataset) | 4176.25 | 115.17 | 728.97 | 126.53 | 10.18 | 6.29 |
| TIN + LG OK (full dataset) | 1351.64♦ | 32.65♦ | 391.09 | 57.51 | 7.22 | 1.37 |
| TIN + OK (full dataset) | 1378.02 | 33.82 | 413.13 | 71.73 | 8.62 | 1.59 |
| TIN + LG OK (split dataset) | 4226.67 | 119.09 | 928.47 | 123.86 | 5.54 | 6.44 |
| *Observed Sample data* |  |  |  |  |  |  |
| Skewness (original dataset) | 24.32 | 22.50 | 1.51 | 1.46 | 0.66 | 4.10 |
| Kurtosis (original dataset) | 803.43 | 668.39 | 6.10 | 4.31 | 0.45 | 42.21 |
| Mean (original dataset) | 337.38 | 13.42 | 451.25 | 66.95 | 8.55 | 1.04 |
| ♦ Indicates the lowest RMSE for that metal | | | | | | |

# Individual biomarker ~ soil model results

## Model summary

Comparing standardized coefficients, in general we found that soil concentration was positively associated with and predicted blood and urine-meal concentrations (Supplemental Figure 2). Not all soil concentration estimates (e.g., closest value) were best at predicted blood or urine-metal level an the dose metrics in general had the greatest number of associations with biomarkers.

| 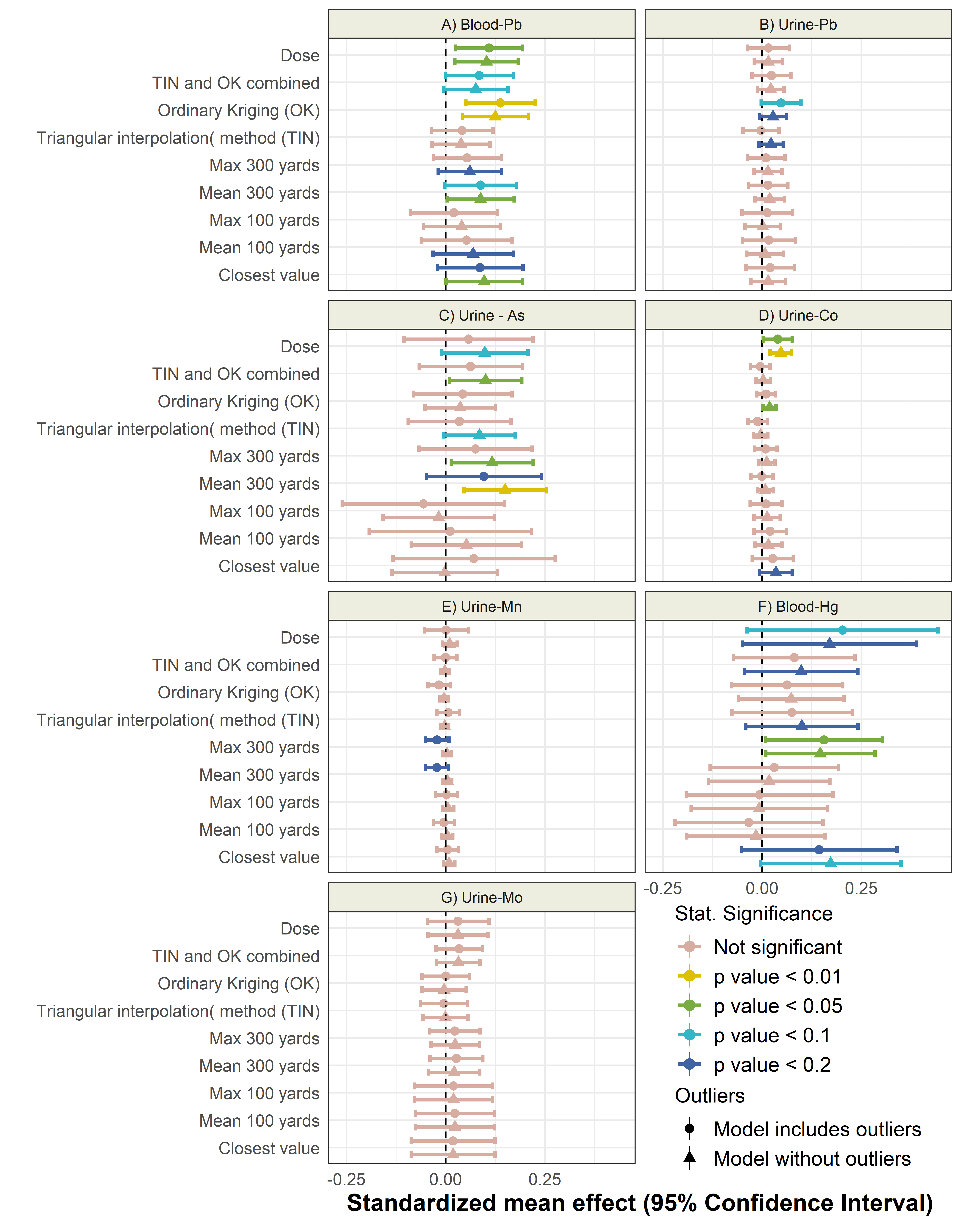 |
| --- |
| **Supplemental Figure 2**: Coefficients and effect sizes for soil-metals as predictors of blood or urine-metal levels. A) Blood-Pb as predicted by soil Pb; higher soil Pb was associated with higher blood-Pb levels for five of the soil measures. B) Urine-Pb as predicted by soil Pb; higher values of the soil-Pb levels as estimated by ordinary Kriging were associated with higher urine-Pb levels in the cohort. C) Urine-As as predicted by soil As; the average soil-As concentration from the observed samples was associated with higher urine-As levels among the cohort. D) Urine-Co as predicted by soil Co; dose calculations for daily intake of soil-Co was associated with higher urine-Co levels in the cohort. E) Urine-Mn as predicted by soil Mn; higher levels of soil-Mn were not associated with higher levels of urine-Mn in the cohort. Greater levels of soil-Mn within 300 yards of the participants’ homes was associated with lower urine-Mn levels F) Blood-Hg as predicted by soil Hg; higher levels of estimated soil-Hg near the homes and daily estimated dose intakes were associated with higher blood-Hg levels in the cohort. G) Urine-Mo as predicted by soil Mo; soil-Mo was not associated with changes in urine-Mo levels. |

## Blood-Lead (Pb)

| Supplemental Table 2: Blood Lead model results | | | | | | | | | |
| --- | --- | --- | --- | --- | --- | --- | --- | --- | --- |
| Predictor name | Closest value (natural log) | Mean 90 m (natural log) | Max 90 m (natural log + 1) | Mean 275 m (natural log) | Max 275 m (natural log + 1) | Triangular irregular network (TIN) (natural log + 1) | Ordinary Kriging (OK) (natural log) | TIN and OK combined (natural log) | Dose: OK (natural log) |
| Model type | Spatial | Spatial | Spatial | Spatial | Spatial | Spatial | Spatial | Spatial | Spatial |
|  | *Estimate (SE)* | *Estimate (SE)* | *Estimate (SE)* | *Estimate (SE)* | *Estimate (SE)* | *Estimate (SE)* | *Estimate (SE)* | *Estimate (SE)* | *Estimate (SE)* |
| Intercept | -0.125 (0.099) | -0.135• (0.1) | -0.145• (0.099) | -0.058 (0.073) | -0.065 (0.073) | -0.108• (0.068) | -0.089• (0.067) | -0.098• (0.067) | -0.099• (0.067) |
| Soil concentration | 0.087• (0.055) | 0.053 (0.058) | 0.021 (0.056) | 0.088. (0.046) | 0.054 (0.044) | 0.041 (0.04) | 0.138*** (0.045) | 0.085• (0.044) | 0.109** (0.043) |
| Race | -0.077 (0.122) | -0.062 (0.121) | -0.053 (0.121) | -0.114 (0.094) | -0.111 (0.094) | -0.162• (0.083) | -0.194** (0.081) | -0.173** (0.082) | -0.175** (0.082) |
| Gender | -0.235** (0.097) | -0.237** (0.098) | -0.234** (0.098) | -0.161** (0.074) | -0.16** (0.075) | -0.13• (0.067) | -0.131** (0.066) | -0.132** (0.067) | -0.116• (0.066) |
| SES | -0.026 (0.06) | -0.03 (0.06) | -0.034 (0.06) | -0.025 (0.045) | -0.032 (0.045) | -0.025 (0.041) | -0.011 (0.04) | -0.018 (0.041) | -0.021 (0.04) |
| Distance to highway | 0.054 (0.053) | 0.058 (0.053) | 0.056 (0.053) | 0.041 (0.043) | 0.04 (0.044) | 0.055• (0.039) | 0.064• (0.038) | 0.055• (0.039) | 0.059• (0.038) |
| BMI | -0.06 (0.05) | -0.064 (0.051) | -0.071• (0.05) | -0.107*** (0.038) | -0.11*** (0.039) | -0.105*** (0.035) | -0.092*** (0.034) | -0.099*** (0.035) | -0.059• (0.039) |
| Age | -0.12** (0.046) | -0.122*** (0.047) | -0.121** (0.047) | -0.095** (0.038) | -0.093** (0.038) | -0.082** (0.034) | -0.083** (0.034) | -0.084** (0.034) | -0.061• (0.035) |
| Parent smoking status | 0.187• (0.11) | 0.194• (0.111) | 0.201• (0.111) | 0.13• (0.085) | 0.135• (0.085) | 0.146• (0.079) | 0.139• (0.077) | 0.139• (0.078) | 0.131• (0.078) |
| HEI total fruit | 0.079• (0.052) | 0.074• (0.052) | 0.071• (0.052) | 0.072• (0.038) | 0.069• (0.039) | 0.063• (0.035) | 0.07** (0.034) | 0.066• (0.034) | 0.072** (0.034) |
| Percent vacant homes | 0.09• (0.062) | 0.102• (0.062) | 0.115• (0.061) | 0.075• (0.049) | 0.095** (0.047) | 0.135*** (0.042) | 0.069• (0.047) | 0.104** (0.046) | 0.108** (0.043) |
| Maximum temperature on day of sample | -0.04 (0.049) | -0.043 (0.05) | -0.039 (0.05) | 0.023 (0.039) | 0.025 (0.039) | -0.005 (0.035) | -0.006 (0.035) | -0.004 (0.035) | -0.004 (0.035) |
| R2 | 0.32 | 0.31 | 0.31 | 0.25 | 0.24 | 0.28 | 0.31 | 0.29 | 0.3 |
| n | 127 | 127 | 127 | 213 | 213 | 249 | 249 | 249 | 249 |
| Moran's I | 2.876*** | 2.792*** | 2.783*** | 2.71*** | 2.668*** | 2.809*** | 2.857*** | 2.861*** | 2.807*** |
| **** p value < 0.01, ** p value < 0.05, * p value < 0.1, . p value < 0.2* | | | | | | | | | |

## Urine-lead (Pb)

| Supplemental Table 3: Urine Lead model results | | | | | | | | | |
| --- | --- | --- | --- | --- | --- | --- | --- | --- | --- |
| Predictor name | Closest value (natural log) | Mean 90 m (natural log) | Max 90 m (natural log + 1) | Mean 275 m (natural log) | Max 275 m (natural log + 1) | Triangular irregular network (TIN) (natural log + 1) | Ordinary Kriging (OK) (natural log) | TIN and OK combined (natural log) | Dose: OK (natural log) |
| Model type | OLS | OLS | OLS | OLS | OLS | OLS | OLS | OLS | OLS |
|  | *Estimate (SE)* | *Estimate (SE)* | *Estimate (SE)* | *Estimate (SE)* | *Estimate (SE)* | *Estimate (SE)* | *Estimate (SE)* | *Estimate (SE)* | *Estimate (SE)* |
| Intercept | 0.371*** (0.056) | 0.37*** (0.056) | 0.368*** (0.056) | 0.399*** (0.04) | 0.398*** (0.04) | 0.399*** (0.04) | 0.405*** (0.039) | 0.401*** (0.04) | 0.4*** (0.04) |
| Soil concentration | 0.02 (0.031) | 0.017 (0.034) | 0.013 (0.033) | 0.015 (0.025) | 0.01 (0.024) | -0.003 (0.023) | 0.047• (0.025) | 0.023 (0.025) | 0.016 (0.027) |
| Race | 0.075 (0.067) | 0.076 (0.067) | 0.079 (0.067) | 0.024 (0.051) | 0.026 (0.051) | 0.019 (0.049) | 0.007 (0.049) | 0.018 (0.049) | 0.019 (0.049) |
| Gender | 0.029 (0.059) | 0.026 (0.06) | 0.026 (0.06) | 0.021 (0.045) | 0.022 (0.045) | 0.007 (0.044) | 0.005 (0.044) | 0.007 (0.044) | 0.009 (0.044) |
| SES | -0.076** (0.033) | -0.077** (0.033) | -0.078** (0.033) | -0.06** (0.025) | -0.062** (0.025) | -0.052** (0.025) | -0.045• (0.025) | -0.048• (0.025) | -0.049• (0.025) |
| Distance to highway | -0.017 (0.03) | -0.014 (0.03) | -0.015 (0.03) | -0.026 (0.023) | -0.026 (0.023) | -0.021 (0.023) | -0.021 (0.022) | -0.024 (0.022) | -0.023 (0.022) |
| BMI | -0.046• (0.031) | -0.047• (0.032) | -0.049• (0.031) | -0.067*** (0.022) | -0.067*** (0.022) | -0.078*** (0.022) | -0.072*** (0.022) | -0.075*** (0.022) | -0.071*** (0.025) |
| Age | -0.035 (0.031) | -0.036 (0.031) | -0.036 (0.031) | -0.034• (0.023) | -0.034• (0.023) | -0.038• (0.023) | -0.039• (0.022) | -0.039• (0.023) | -0.035• (0.023) |
| Parent smoking status | -0.006 (0.064) | -0.003 (0.064) | -0.002 (0.064) | -0.025 (0.048) | -0.025 (0.048) | -0.014 (0.049) | -0.014 (0.049) | -0.016 (0.049) | -0.017 (0.049) |
| HEI total fruit | -0.003 (0.031) | -0.005 (0.031) | -0.005 (0.031) | 0.012 (0.022) | 0.011 (0.022) | 0.022 (0.022) | 0.025 (0.022) | 0.022 (0.022) | 0.024 (0.022) |
| Percent vacant homes | 0.051• (0.034) | 0.051• (0.036) | 0.053• (0.035) | 0.048• (0.027) | 0.052** (0.026) | 0.057** (0.025) | 0.03 (0.028) | 0.046• (0.027) | 0.051** (0.026) |
| Maximum temperature on day of sample | -0.02 (0.03) | -0.022 (0.031) | -0.021 (0.031) | -0.002 (0.023) | -0.002 (0.023) | -0.004 (0.022) | -0.003 (0.022) | -0.002 (0.022) | -0.003 (0.022) |
| R2 | 0.19 | 0.19 | 0.18 | 0.17 | 0.17 | 0.16 | 0.17 | 0.16 | 0.16 |
| n | 109 | 109 | 109 | 181 | 181 | 207 | 207 | 207 | 207 |
| Moran's I | -1.058 | -1.054 | -1.058 | -0.885 | -0.914 | -0.722 | -0.54 | -0.648 | -0.644 |
| **** p value < 0.01, ** p value < 0.05, * p value < 0.1, . p value < 0.2* | | | | | | | | | |

## Arsenic (As)

| Supplemental Table 4: Urine arsenic model results | | | | | | | | | |
| --- | --- | --- | --- | --- | --- | --- | --- | --- | --- |
| Predictor name | Closest value (natural log + 1) | Mean 100 yards (natural log) | Max 100 yards (natural log + 1) | Mean 300 yards (natural log) | Max 300 yards (natural log + 1) | Triangular irregular network (TIN) (natural log + 1) | Ordinary Kriging (OK) (not log transformed) | TIN and OK combined (natural log) | Dose: TIN and OK combined (natural log) |
| Model type | Spatial | OLS | OLS | Spatial | Spatial | Spatial | Spatial | Spatial | Spatial |
|  | *Estimate (SE)* | *Estimate (SE)* | *Estimate (SE)* | *Estimate (SE)* | *Estimate (SE)* | *Estimate (SE)* | *Estimate (SE)* | *Estimate (SE)* | *Estimate (SE)* |
| Intercept | 2.344*** (0.196) | 2.323*** (0.181) | 2.308*** (0.179) | 2.298*** (0.12) | 2.294*** (0.12) | 2.3*** (0.115) | 2.31*** (0.115) | 2.301*** (0.114) | 2.304*** (0.114) |
| Soil concentration | 0.071 (0.104) | 0.011 (0.104) | -0.056 (0.104) | 0.096• (0.074) | 0.075 (0.073) | 0.035 (0.066) | 0.042 (0.063) | 0.063 (0.066) | 0.057 (0.083) |
| Race | -0.203 (0.235) | -0.167 (0.211) | -0.163 (0.211) | -0.07 (0.15) | -0.078 (0.15) | -0.013 (0.138) | -0.022 (0.136) | -0.003 (0.138) | -0.023 (0.136) |
| Gender | 0.188 (0.205) | 0.129 (0.203) | 0.162 (0.204) | -0.008 (0.132) | -0.001 (0.132) | -0.046 (0.125) | -0.057 (0.126) | -0.05 (0.125) | -0.037 (0.125) |
| SES | 0.071 (0.12) | 0.005 (0.112) | 0.01 (0.112) | -0.065 (0.076) | -0.074 (0.076) | -0.133• (0.071) | -0.136• (0.071) | -0.129• (0.071) | -0.130• (0.071) |
| Distance to highway | -0.013 (0.114) | 0.027 (0.107) | 0.007 (0.107) | 0.058 (0.07) | 0.052 (0.07) | 0.06 (0.066) | 0.068 (0.066) | 0.062 (0.065) | 0.062 (0.065) |
| BMI | 0.027 (0.111) | 0.103 (0.103) | 0.101 (0.102) | 0.002 (0.068) | -0.001 (0.068) | -0.06 (0.063) | -0.058 (0.063) | -0.057 (0.063) | -0.032 (0.075) |
| Age | -0.061 (0.1) | -0.118 (0.1) | -0.115 (0.1) | -0.100• (0.066) | -0.098• (0.066) | -0.061 (0.062) | -0.06 (0.062) | -0.064 (0.063) | -0.044 (0.066) |
| Parent smoking status | -0.106 (0.22) | -0.116 (0.206) | -0.113 (0.206) | -0.094 (0.143) | -0.086 (0.143) | -0.138 (0.139) | -0.143 (0.139) | -0.145 (0.139) | -0.148 (0.14) |
| Maximum temperature on day of sample | 0.152• (0.102) | 0.101 (0.1) | 0.112 (0.1) | 0.093• (0.068) | 0.093• (0.068) | 0.095• (0.064) | 0.092• (0.064) | 0.096• (0.064) | 0.096• (0.064) |
| R2 | 0.09 | 0.06 | 0.06 | 0.05 | 0.05 | 0.04 | 0.04 | 0.05 | 0.04 |
| n | 101 | 111 | 111 | 203 | 203 | 233 | 233 | 233 | 233 |
| Moran's I | 1.135• | 0.622 | 0.691 | 1.124• | 1.073• | 0.958• | 1.059• | 0.92• | 0.919• |
| **** p value < 0.01, ** p value < 0.05, * p value < 0.1, • p value < 0.2* | | | | | | | | | |

## Cobalt (Co)

| Supplemental Table 5: Urine Co model results | | | | | | | | | |
| --- | --- | --- | --- | --- | --- | --- | --- | --- | --- |
| Predictor name | Closest value (natural log) | Mean 90 m (natural log + 1) | Max 90 m (natural log + 1) | Mean 275 m (not log transformed) | Max 275 m (natural log + 1) | Triangular irregular network (TIN) (natural log + 1) | Ordinary Kriging (OK) (not log transformed) | TIN and OK combined (natural log) | Dose: TIN (natural log) |
| Model type | Spatial | Spatial | Spatial | Spatial | Spatial | Spatial | Spatial | Spatial | Spatial |
|  | *Estimate (SE)* | *Estimate (SE)* | *Estimate (SE)* | *Estimate (SE)* | *Estimate (SE)* | *Estimate (SE)* | *Estimate (SE)* | *Estimate (SE)* | *Estimate (SE)* |
| Intercept | 0.372*** (0.058) | 0.346*** (0.048) | 0.345*** (0.048) | 0.357*** (0.03) | 0.358*** (0.03) | 0.367*** (0.028) | 0.368*** (0.028) | 0.365*** (0.028) | 0.357*** (0.032) |
| Soil concentration | 0.027 (0.026) | 0.02 (0.021) | 0.01 (0.021) | -0.001 (0.014) | 0.009 (0.014) | -0.012 (0.013) | 0.009 (0.012) | -0.005 (0.013) | 0.039** (0.019) |
| Race | 0.068 (0.055) | 0.088• (0.045) | 0.087• (0.045) | 0.079*** (0.03) | 0.077** (0.03) | 0.049• (0.027) | 0.053** (0.026) | 0.052• (0.026) | 0.051• (0.032) |
| Gender | 0.094• (0.054) | 0.068• (0.043) | 0.064• (0.043) | 0.067** (0.026) | 0.067** (0.026) | 0.063*** (0.024) | 0.062** (0.024) | 0.063*** (0.024) | 0.064** (0.027) |
| SES | -0.032 (0.028) | -0.026 (0.024) | -0.027 (0.024) | -0.016 (0.015) | -0.017 (0.015) | -0.016 (0.014) | -0.015 (0.014) | -0.015 (0.014) | -0.015 (0.016) |
| Distance to highway | 0.034• (0.024) | 0.031• (0.021) | 0.033• (0.021) | 0.009 (0.014) | 0.007 (0.014) | 0.016 (0.013) | 0.012 (0.013) | 0.014 (0.013) | 0.011 (0.014) |
| BMI | -0.072** (0.029) | -0.051** (0.022) | -0.051** (0.022) | -0.027** (0.013) | -0.027** (0.013) | -0.022• (0.012) | -0.022• (0.012) | -0.022• (0.012) | -0.02 (0.017) |
| Age | 0.013 (0.025) | 0.011 (0.021) | 0.012 (0.021) | 0.023• (0.013) | 0.024• (0.013) | 0.022• (0.012) | 0.02• (0.012) | 0.021• (0.012) | 0.038*** (0.014) |
| Parent smoking status | -0.008 (0.057) | 0.003 (0.047) | 0.002 (0.047) | -0.05• (0.029) | -0.048• (0.029) | -0.047• (0.027) | -0.049• (0.027) | -0.047• (0.027) | -0.063** (0.03) |
| Maximum temperature on day of sample | 0.001 (0.026) | 0.012 (0.022) | 0.014 (0.022) | 0.006 (0.014) | 0.005 (0.014) | 0.003 (0.012) | 0.002 (0.012) | 0.003 (0.012) | 0.007 (0.014) |
| R2 | 0.22 | 0.18 | 0.17 | 0.12 | 0.13 | 0.1 | 0.1 | 0.1 | 0.18 |
| n | 69 | 93 | 93 | 201 | 201 | 233 | 233 | 233 | 165 |
| Moran's I | 0.895• | 0.962• | 0.944• | 1.275• | 1.331• | 1.42• | 1.368• | 1.423• | 1.72** |
| **** p value < 0.01, ** p value < 0.05, * p value < 0.1, . p value < 0.2* | | | | | | | | | |

## Manganese (Mn)

| Supplemental Table 6: Urine Mn model results | | | | | | | | | |
| --- | --- | --- | --- | --- | --- | --- | --- | --- | --- |
| Predictor name | Closest value (natural log) | Mean 90 m (natural log) | Max 90 m (natural log + 1) | Mean 275 m (not log transformed) | Max 275 m (natural log + 1) | Triangular irregular network (TIN) (natural log + 1) | Ordinary Kriging (OK) (not log transformed) | TIN and OK combined (natural log) | Dose: LG OK (natural log) |
| Model type | OLS | OLS | OLS | OLS | OLS | OLS | OLS | OLS | OLS |
|  | *Estimate (SE)* | *Estimate (SE)* | *Estimate (SE)* | *Estimate (SE)* | *Estimate (SE)* | *Estimate (SE)* | *Estimate (SE)* | *Estimate (SE)* | *Estimate (SE)* |
| Intercept | 0.157*** (0.031) | 0.155*** (0.031) | 0.155*** (0.031) | 0.196*** (0.032) | 0.192*** (0.032) | 0.192*** (0.032) | 0.194*** (0.032) | 0.193*** (0.032) | 0.193*** (0.032) |
| Soil concentration | 0.004 (0.014) | -0.005 (0.014) | 0.002 (0.014) | -0.022• (0.015) | -0.022• (0.015) | 0.006 (0.015) | -0.016 (0.014) | -0.004 (0.014) | 0.002 (0.028) |
| Race | 0.008 (0.03) | 0.009 (0.03) | 0.009 (0.03) | -0.023 (0.032) | -0.018 (0.032) | -0.009 (0.031) | -0.012 (0.03) | -0.011 (0.03) | -0.012 (0.03) |
| Gender | 0.036• (0.028) | 0.036• (0.028) | 0.036 (0.028) | 0.011 (0.029) | 0.014 (0.029) | 0.01 (0.028) | 0.01 (0.028) | 0.009 (0.028) | 0.01 (0.029) |
| SES | 0.026• (0.016) | 0.028• (0.016) | 0.027• (0.016) | 0.035** (0.016) | 0.038** (0.017) | 0.032** (0.016) | 0.033** (0.016) | 0.031• (0.016) | 0.031** (0.016) |
| Distance to highway | -0.007 (0.014) | -0.009 (0.014) | -0.008 (0.014) | -0.028• (0.015) | -0.027• (0.015) | -0.025• (0.015) | -0.021• (0.014) | -0.024• (0.015) | -0.024• (0.014) |
| BMI | 0.026• (0.014) | 0.027• (0.014) | 0.027• (0.014) | 0.035** (0.015) | 0.033** (0.015) | 0.021• (0.014) | 0.02• (0.014) | 0.021• (0.014) | 0.022 (0.024) |
| Age | -0.041*** (0.014) | -0.041*** (0.014) | -0.041*** (0.014) | -0.024• (0.015) | -0.023• (0.015) | -0.023• (0.014) | -0.022• (0.014) | -0.022• (0.014) | -0.022 (0.018) |
| Parent smoking status | 0.006 (0.029) | 0.006 (0.029) | 0.006 (0.029) | -0.013 (0.031) | -0.01 (0.031) | -0.011 (0.031) | -0.012 (0.031) | -0.011 (0.031) | -0.011 (0.031) |
| Maximum temperature on day of sample | 0.009 (0.014) | 0.01 (0.014) | 0.009 (0.014) | 0.009 (0.015) | 0.009 (0.014) | 0.006 (0.014) | 0.006 (0.014) | 0.006 (0.014) | 0.006 (0.014) |
| R2 | 0.14 | 0.14 | 0.14 | 0.07 | 0.07 | 0.04 | 0.05 | 0.04 | 0.04 |
| n | 117 | 117 | 117 | 203 | 203 | 233 | 233 | 233 | 233 |
| Moran's I | -0.579 | -0.545 | -0.569 | -1.295 | -1.32 | -0.822 | -0.831 | -0.748 | -0.796 |
| **** p value < 0.01, ** p value < 0.05, * p value < 0.1, . p value < 0.2* | | | | | | | | | |

## Mercury (Hg)

| Supplemental Table 7: Blood Mercury model results | | | | | | | | | |
| --- | --- | --- | --- | --- | --- | --- | --- | --- | --- |
| Soil concentration predictor name | Closest value (natural log + 1) | Mean 90 m (natural log) | Max 90 m (natural log + 1) | Mean 275 m (not log transformed) | Max 275 m (natural log + 1) | Triangular irregular network (TIN) (natural log + 1) | Ordinary Kriging (OK) (not log transformed) | TIN and OK combined (natural log) | Dose: OK (natural log) |
| Model type | Spatial | Spatial | Spatial | Spatial | Spatial | Spatial | Spatial | Spatial | Spatial |
|  | *Estimate (SE)* | *Estimate (SE)* | *Estimate (SE)* | *Estimate (SE)* | *Estimate (SE)* | *Estimate (SE)* | *Estimate (SE)* | *Estimate (SE)* | Est. (SE) |
| Intercept | 8.239• (4.461) | 7.529• (4.314) | 7.7• (4.304) | 3.864 (3.374) | 3.77 (3.36) | 4.019 (3.261) | 4.355• (3.269) | 4.051 (3.26) | 4.421• (3.26) |
| Soil concentration | 0.143• (0.1) | -0.034 (0.095) | -0.007 (0.094) | 0.03 (0.083) | 0.155** (0.075) | 0.075 (0.077) | 0.063 (0.072) | 0.081 (0.078) | 0.203* (0.123) |
| Race | -0.041 (0.231) | -0.088 (0.229) | -0.098 (0.228) | -0.173 (0.18) | -0.119 (0.177) | -0.094 (0.164) | -0.126 (0.162) | -0.095 (0.163) | -0.14 (0.161) |
| Gender | -0.132 (0.2) | -0.177 (0.189) | -0.176 (0.189) | 0.027 (0.144) | 0.046 (0.143) | 0.104 (0.139) | 0.097 (0.139) | 0.106 (0.139) | 0.148 (0.141) |
| SES | 0.124 (0.12) | 0.106 (0.115) | 0.114 (0.113) | 0.036 (0.086) | 0.032 (0.084) | -0.02 (0.081) | -0.024 (0.081) | -0.018 (0.081) | -0.019 (0.08) |
| Distance to highway | -0.045 (0.099) | -0.002 (0.096) | -0.006 (0.095) | -0.022 (0.081) | -0.024 (0.079) | -0.038 (0.077) | -0.024 (0.076) | -0.041 (0.078) | -0.022 (0.075) |
| BMI | 0.137 (0.109) | 0.147• (0.1) | 0.149• (0.1) | 0.09 (0.075) | 0.1• (0.074) | 0.02 (0.072) | 0.019 (0.072) | 0.022 (0.072) | 0.15. (0.108) |
| Age | -0.089 (0.105) | -0.093 (0.099) | -0.096 (0.099) | -0.021 (0.075) | -0.025 (0.075) | -0.001 (0.075) | -0.003 (0.075) | -0.003 (0.075) | 0.068 (0.083) |
| Parent smoking status | -0.062 (0.21) | -0.004 (0.202) | -0.01 (0.202) | 0.026 (0.163) | 0.01 (0.159) | -0.028 (0.156) | -0.02 (0.156) | -0.029 (0.156) | -0.038 (0.156) |
| RSEI air concentration | 0.982** (0.391) | 0.915** (0.377) | 0.93** (0.376) | 0.54• (0.297) | 0.53• (0.296) | 0.548• (0.287) | 0.574** (0.288) | 0.551• (0.287) | 0.58** (0.287) |
| HEI sodium | -0.052• (0.038) | -0.052• (0.037) | -0.051• (0.037) | -0.046• (0.026) | -0.049• (0.026) | -0.028 (0.025) | -0.027 (0.025) | -0.028 (0.025) | -0.03 (0.025) |
| HEI total protein | 0.335*** (0.104) | 0.326*** (0.094) | 0.327*** (0.094) | 0.19*** (0.065) | 0.189*** (0.064) | 0.164*** (0.061) | 0.158*** (0.06) | 0.164*** (0.061) | 0.164*** (0.06) |
| Maximum temperature on day of sample | 0.063 (0.103) | 0.07 (0.098) | 0.068 (0.097) | 0.052 (0.077) | 0.034 (0.076) | 0.011 (0.073) | 0.003 (0.073) | 0.009 (0.073) | <0.001 (0.073) |
| R2 | 0.24 | 0.24 | 0.24 | 0.14 | 0.15 | 0.08 | 0.08 | 0.08 | 0.09 |
| n | 111 | 121 | 121 | 209 | 209 | 244 | 244 | 244 | 244 |
| Moran's I | 0.915• | 1.287• | 1.283• | 2.026** | 1.654** | 1.379• | 1.446• | 1.371• | 1.361• |
| **** p value < 0.01, ** p value < 0.05, * p value < 0.1, • p value < 0.2* | | | | | | | | | |

## Molybdenum

| Supplemental Table 8: Urine Mo model results | | | | | | | | | |
| --- | --- | --- | --- | --- | --- | --- | --- | --- | --- |
| Predictor name | Closest value (natural log + 1) | Mean 90 m (natural log + 1) | Max 90 m (natural log + 1) | Mean 275 m (natural log + 1) | Max 275 m (natural log + 1) | Triangular irregular network (TIN) (natural log + 1) | Ordinary Kriging (OK) (not log transformed) | TIN and OK combined (natural log) | Dose: TIN (natural log) |
| Model type | OLS | OLS | OLS | Spatial | Spatial | Spatial | Spatial | Spatial | Spatial |
|  | *Estimate (SE)* | *Estimate (SE)* | *Estimate (SE)* | *Estimate (SE)* | *Estimate (SE)* | *Estimate (SE)* | *Estimate (SE)* | *Estimate (SE)* | *Estimate (SE)* |
| Intercept | 4.243*** (0.137) | 4.174*** (0.115) | 4.171*** (0.115) | 4.079*** (0.068) | 4.076*** (0.068) | 4.084*** (0.066) | 4.085*** (0.066) | 4.087*** (0.066) | 4.081*** (0.087) |
| Soil concentration | 0.019 (0.054) | 0.023 (0.051) | 0.019 (0.05) | 0.027 (0.034) | 0.023 (0.032) | -0.005 (0.03) | -0.0004 (0.03) | 0.034 (0.03) | 0.031 (0.04) |
| Race | -0.041 (0.123) | 0 (0.103) | 0 (0.105) | 0.16** (0.07) | 0.167** (0.069) | 0.181*** (0.063) | 0.182*** (0.063) | 0.181*** (0.062) | 0.145• (0.087) |
| Gender | -0.162• (0.103) | -0.107 (0.092) | -0.11 (0.093) | -0.08• (0.06) | -0.082• (0.06) | -0.097• (0.057) | -0.096• (0.057) | -0.093• (0.057) | -0.063 (0.073) |
| SES | 0.004 (0.056) | -0.003 (0.052) | -0.004 (0.052) | -0.03 (0.034) | -0.03 (0.034) | -0.023 (0.033) | -0.023 (0.032) | -0.019 (0.032) | -0.013 (0.043) |
| Distance to highway | 0.006 (0.057) | 0.004 (0.047) | 0.004 (0.048) | 0.023 (0.034) | 0.018 (0.033) | 0.011 (0.03) | 0.011 (0.032) | 0.018 (0.031) | 0.032 (0.039) |
| BMI | -0.086• (0.058) | -0.092• (0.051) | -0.093• (0.052) | -0.067** (0.031) | -0.066** (0.031) | -0.054• (0.029) | -0.054• (0.029) | -0.056** (0.029) | -0.042 (0.04) |
| Age | -0.023 (0.054) | -0.057 (0.048) | -0.054 (0.048) | -0.034 (0.03) | -0.035 (0.03) | -0.058** (0.028) | -0.059** (0.028) | -0.061** (0.028) | -0.045 (0.036) |
| Parent smoking status | -0.051 (0.113) | -0.078 (0.1) | -0.075 (0.1) | -0.065 (0.066) | -0.064 (0.066) | -0.061 (0.064) | -0.062 (0.063) | -0.062 (0.063) | -0.083 (0.082) |
| Maximum temperature on day of sample | 0.049 (0.057) | 0.071• (0.049) | 0.073• (0.049) | 0.049• (0.031) | 0.049• (0.031) | 0.049• (0.03) | 0.048• (0.029) | 0.046• (0.029) | 0.069• (0.04) |
| R2 | 0.08 | 0.1 | 0.09 | 0.11 | 0.11 | 0.11 | 0.11 | 0.12 | 0.16 |
| n | 71 | 87 | 87 | 202 | 202 | 233 | 233 | 233 | 143 |
| Moran's I | 0.516 | 0.329 | 0.337 | 1.371• | 1.334• | 1.197• | 1.208• | 1.152• | 1.783** |
| **** p value < 0.01, ** p value < 0.05, * p value < 0.1, . p value < 0.2* | | | | | | | | | |

# Sensitivity analysis for time spent in home

The log-transformed models had the best linear fit between the soil exposure variables and the blood/urine metal outcome variables, however, we also fit models with the data on their original scales, unstandardized. We did this to contribute estimates for the change in soil concentration and its association for the change in blood/urine metal levels for the soil metrics that were statistically significant for blood-Pb, and urine-Co. Children living in the same home less than 5 years on average had 2.7 µg/dL higher blood-Pb levels for every 1000 ppm of soil-Pb with 12 percent variance explained (Supplemental Figure 3a). If the child lived in their home more than five years, they had 1.7 µg/dL higher blood-Pb levels for every 1000 µg/g of soil-Pb with 3.6 variance explained by soil-Pb (Supplemental Figure 3a). Children living in their same home for less than 5 years had 0.95 µg/dL higher blood-Pb for every 1 additional µg/kg/day of soil-Pb exposure with 11 percent variance explained by soil-Pb dose (Figure 4b). Children living in their same home for more than 5 years had 0.37 µg/dL higher blood-Pb for every 1 additional µg/kg/day of soil-Pb exposure with 7.4 percent variance explained by soil-Pb (Supplemental Figure 3b). For Co, children living in the same home for less than 5 years did not have higher urine-Co even when exposed to more soil-Co (Figure 4c), however, if the child reported living in the same home more than 5 years, they had 2.7 µg/g creatinine higher adjusted urine-Co levels for every 90 mg/kg more potential soil-Co exposure with 5.1 variance explained by soil-Co (Supplemental Figure 3c). For Co exposure dose, Children living in their home less than 5 years had 0.76 µg/g creatinine higher creatinine adjusted urine-Co levels for every 1 µg/kg/day of soil-Co exposure with about 1 percent of the variance explained by soil-Co (Supplemental Figure 3d). If the child reported living in their residence more than 5 years, then on average, the child had 2 µg/g creatinine higher creatinine adjusted urine-Co levels for ever 1 µg/kg/day of soil-Co exposure with 30 percent variance explained by soil-Co (Supplemental Figure 3d).

| 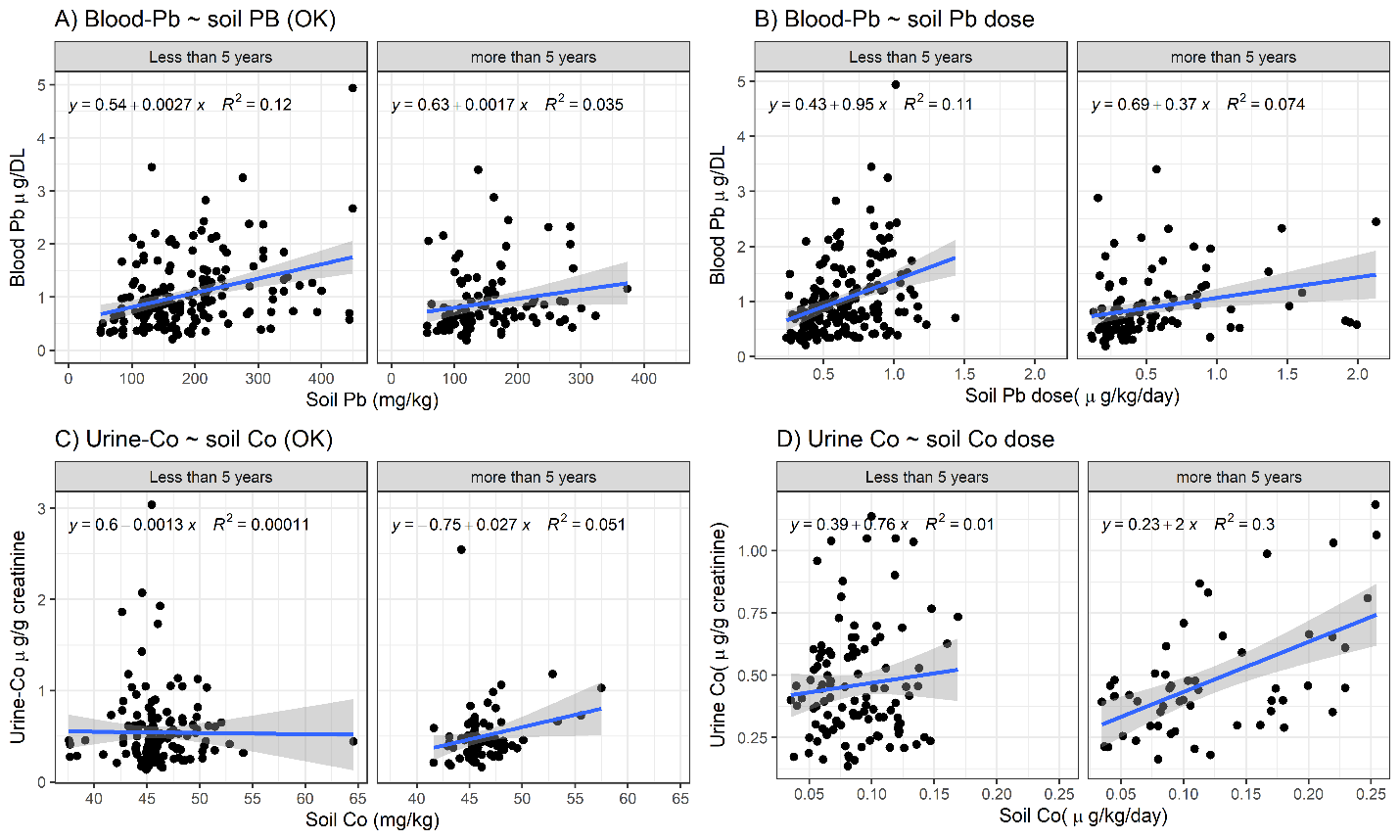 |
| --- |
| **Supplemental Figure 3:** A) Linear regression for blood-Pb and OK soil-Pb stratified by the number of years the participant reported living in their current residence (4 outliers removed). B) Linear regression results for blood-Pb and soil-Pb exposure dose (6 outliers removed). C) Linear regression results for urine-Co and soil OK soil-Co stratified by the number of years the participant reported living in their current residence (no outliers removed). D) Linear regression results for urine-Co and soil-Co exposure dose (5 outliers removed). |

# Descriptive statistics for the estimated dose of exposure for the cohort

| Table 9: Dose estimate descriptive statistics for each soil-metal concentration | | | | | | |
| --- | --- | --- | --- | --- | --- | --- |
| *Variable* | *n* | *Mean* | *sd* | *Min* | *Median* | *Max* |
| Soil-Pb dose (mg/kg/day) | 281 | 0.000617 | 0.000397 | 0.000110 | 0.000540 | 0.004352 |
| Soil-As dose (mg/kg/day) | 281 | 0.000030 | 0.000013 | 0.000008 | 0.000029 | 0.000109 |
| Soil-Mo dose (mg/kg/day) | 176 | 0.000002 | 0.000001 | 0.000000 | 0.000001 | 0.000010 |
| Soil-Co dose (mg/kg/day) | 201 | 0.000103 | 0.000055 | 0.000035 | 0.000090 | 0.000383 |
| Soil-Hg dose (mg/kg/day) | 281 | 0.000015 | 0.000005 | 0.000006 | 0.000015 | 0.000031 |
| Soil-Mn dose (mg/kg/day) | 281 | 0.000743 | 0.000223 | 0.000294 | 0.000727 | 0.001324 |

# References

(1) Griffith, D. A.; Johnson, D. L.; Hunt, A. The Geographic Distribution of Metals in Urban Soils: The Case of Syracuse, NY. *GeoJournal* **2009**, *74* (4), 275–291. https://doi.org/10.1007/s10708-008-9233-x.
